# A putative hepatitis B virus sequence motif associated with hepatocellular carcinoma in South African adults

**DOI:** 10.1016/j.aohep.2024.101763

**Published:** 2025-02-20

**Authors:** Tongai G. Maponga, Anna L. McNaughton, Cori Campbell, Mariateresa de Cesare, Jolynne Mokaya, Sheila F. Lumley, David Bonsall, Camilla LC Ip, Haiting Chai, Christo Van Rensburg, Richard H. Glashoff, Elizabeth Waddilove, Wolfgang Preiser, Jason T. Blackard, M. Azim Ansari, Anna Kramvis, Monique I. Andersson, Philippa C Matthews

**Affiliations:** a Division of Medical Virology, Stellenbosch University, Faculty of Medicine and Health Sciences, Tygerberg, Cape Town, South Africa; b National Health Laboratory Service, Tygerberg Business Unit, Tygerberg, Cape Town, South Africa; c Nuffield Department of Medicine, University of Oxford, Oxford, UK; d Parasites and Microbes, Wellcome Sanger Institute, Hinxton, UK; e Department of Infectious Diseases and Microbiology, Oxford University Hospitals NHS Foundation Trust, John Radcliffe Hospital, Headley Way, Oxford OX3 9DU, UK; f Big Data Institute, Old Road, Oxford OX3 7FZ, UK; g Division of Gastroenterology, Stellenbosch University, Faculty of Medicine and Health Sciences, Tygerberg, Cape Town, South Africa; h Immunology Unit, Division of Medical Microbiology, Stellenbosch University, Faculty of Medicine and Health Sciences, Tygerberg, Cape Town, South Africa; i The Francis Crick Institute, 1 Midland Road, London NW1 1AT, UK; j University of Cincinnati College of Medicine, Cincinnati, OH, USA; k Hepatitis Virus Diversity Research Unit, Department of Internal Medicine, University of the Witwatersrand, 7 York Road, Parktown, 2193 Johannesburg, South Africa; l Division of Infection and Immunity, University College London, Gower Street, London WC1E 6BT, UK; m Department of Infectious Diseases, University College London Hospital, Euston Road, London NW1 2BU, UK

**Keywords:** Hepatitis B virus, Hepatocellular carcinoma, Liver cancer, Africa, Whole genome sequencing

## Abstract

**Introduction and Objectives:**

Chronic hepatitis B virus (HBV) infection is a major risk factor for hepatocellular carcinoma (HCC). In African populations, HCC frequently presents at an advanced stage with poor outcomes. We applied whole genome sequencing (WGS) to compare HBV genomes in individuals with and without HCC.

**Materials and Methods:**

We identified adults with HBV infection, with and without complicating HCC, in Cape Town, South Africa. We generated HBV WGS using pan-genotypic probe-based enrichment followed by Illumina sequencing.

**Results:**

Compared to the non-HCC group, HCC patients were more likely to be male (*p* < 0.0001), older (*p* = 0.01), HIV-negative (*p* = 0.006), and have higher HBV viral loads (*p* < 0.0001). Among 19 HCC and 12 non-HCC patients for whom WGS was obtained, genotype A dominated (74 %), of which 96 % were subgenotype A1. PreS2 deletions (Δ38−55) were enriched in HBV sequences from HCC patients (*n* = 7). The sequence motif most strongly associated with HCC comprised either a deletion or polymorphism at site T53 in PreS2 − collectively coined ‘non-T53’ − together with a basal core promoter (BCP) mutation G1764A (AUROC = 0.79).

**Conclusions:**

In this setting, HBV sequence polymorphisms and deletions are associated with HCC, and ‘non-T53 + G1764A’ represents a putative signature motif for HCC. Additional investigations are needed to disaggregate the impact of other demographic, clinical, and environmental influences, to ascertain the extent to which viral polymorphisms contribute to oncogenesis, and to determine whether HBV sequence is a useful biomarker for HCC risk stratification.

## Abbreviations

AUROC: Area Under the Receiver Operating Curve
BCP: Basal Core Promoter
BEAST: Bayesian Evolutionary Analysis by Sampling Trees
bp: Base Pairs
DNA: Deoxy-ribonucleic Acid
ER: Endoplasmic Reticulum
Genbank: Genetic Sequence Database
HIV: Human Immunodeficiency Virus
HBV: Hepatitis B Virus
HCC: Hepatocellular Carcinoma
IU/mL: International Units per Milliliter
MCMC: Markov Chain Monte Carlo
nt: Nucleotide
PRJEB: Project Identifier in European Nucleotide Archive (ENA)
STATA: Statistical Software for Data Science
VL: Viral Load
WHO: World Health Organization
WGS: Whole Genome Sequencing
X gene: Hepatitis B Virus X gene

## 1 Introduction

In the World Health Organization (WHO) Africa region, an estimated 65 million individuals are living with chronic hepatitis B virus (HBV) infection [1,2]. The high incidence of hepatocellular carcinoma (HCC) in Africa (63,000 cases per annum in 2018) [3,4] reflects the prevalence and distribution of HBV infection [5,6]. HBV-associated HCC in African populations affects adults in early and middle life, who typically present with advanced malignancy, leading to high mortality, with substantial individual and societal impact [7,8]. International targets that aim to eliminate the public health threat of HBV infection include HCC as an important area of focus [9].

Multiple HBV genotypes circulate in Africa, including genotypes A, D and E, with genotypes A and E potentially being of particular significance in contributing to HCC incidence [10]. In Southern Africa, infection with subgenotype A1 HBV has a strong association with HCC [11,12]. Malignant transformation can occur as a result of chronic inflammatory/fibrotic liver disease, HBV DNA integration into the host genome, cell stress caused by the accumulation of aberrant viral proteins [13], and/or a direct influence of HBV genes (particularly the *X* gene) [14]. Specific viral polymorphisms have been associated with HCC, including truncated genes, pre-core insertions/deletions (‘indels’), and basal core promoter (BCP) mutations [15–17]. Such sequence changes can be used potentially to infer disease risk or prognosis [18–20]. However, further work is needed to better describe the mutational landscape of HBV in diverse viral genotypes, advance insights into specific associations between viral sequence polymorphisms and HCC, and determine their mechanistic impact. Evaluation is needed to determine whether viral sequence can inform clinical risk assessment, surveillance or interventions.

We performed HBV sequence analysis from South African adults to explore viral sequence polymorphisms in those with and without HCC.

## 2 Material and Methods

### 2.1 Study samples

We retrospectively drew on banked serum samples from adults with a confirmed diagnosis of chronic HBV infection, in cohorts with and without HCC; the clinical and epidemiological features of these have been previously published (see [21] and [22], respectively) (Suppl methods 1; Suppl Fig. 1).

### 2.2 Illumina sequencing, genome assembly, and phylogenetic analysis

We generated HBV sequence data based on an adapted version of a published Illumina protocol [23] (Suppl methods 2). HBV reads were mapped to reference sequences (genotypes A-I) prior to generating consensus WGS. A standard HBV reference strain (Genbank accession X02763, genotype A) was used for numbering positions in the genome.

For phylogenetic analysis, we aligned nucleotide alignments using new sequence data from this cohort, 61 full-length South African sequences from Genbank, and genotype reference sequences [24] using Clustal X 2.1 [25]. We performed phylogenetic inference using a Bayesian Markov Chain Monte Carlo (MCMC) approach as implemented in the Bayesian Evolutionary Analysis by Sampling Trees (BEAST) version 1.10.4 program (Suppl methods 3).

### 2.3 Analysis

We identified HBV polymorphisms that have been previously associated with HCC [14,26–29] (Table 1), and explored the association between polymorphisms and HCC using area under the receiver-operating curve (AUROC). The frequency of deletions at each site was compared between HCC and non-HCC sequences. Statistical analyses were performed using GraphPad Prism v10 and STATA v17.0.

### 2.4 Ethical statement

Ethical approval was granted from the health research ethics committee at the University of Stellenbosch (S13/04/072 and N11/09/284).

## 3 Results

### 3.1 HBV-associated HCC with male sex and higher HBV viral loads

We identified samples from 161 adults with chronic HBV infection of whom 68 had HCC and 93 not. Compared to the non-HCC group, those with HCC were more likely to be male (81 % vs. 46 %, respectively, *p* < 0.0001), older (median 41 vs. 36 years, *p* = 0.01), and had higher HBV DNA viral loads (median 5.2 vs 3.5 log_10_ IU/mL, *p* < 0.0001). HIV coinfection was present in 18 of 65 HCC cases (3 had no documented test result) and 46 of 93 non-HCC cases (27.8 % vs 49.5 %; *p* = 0.006) (Suppl Table 1).

We were able to undertake WGS HBV sequencing from 19 samples from the HCC group and 14 from the non-HCC group (Suppl Methods 2), with sufficient reads to construct WGS assemblies from 31 of these (19 HCC and 12 non-HCC (Table 1; Suppl Fig. 2)). Sequence data can be accessed in GenBank project PRJEB71107.

Overall, genotype A predominated, accounting for 23 of 31 (74.2 %), of which 22 were subgenotype A1. There was no difference in genotype distribution between HCC and non-HCC groups (Suppl Fig. 1; Suppl Tables 2 and 3; Suppl Results 1). HBV sequences from HCC and non-HCC groups were interspersed with other South African HBV sequences, suggesting there was no particular viral lineage associated with the development of HCC (Suppl Fig. 3).

### 3.2 Deletions and substitutions in HBV Pre-S2 significantly enriched in HCC

**Fig. 1:**
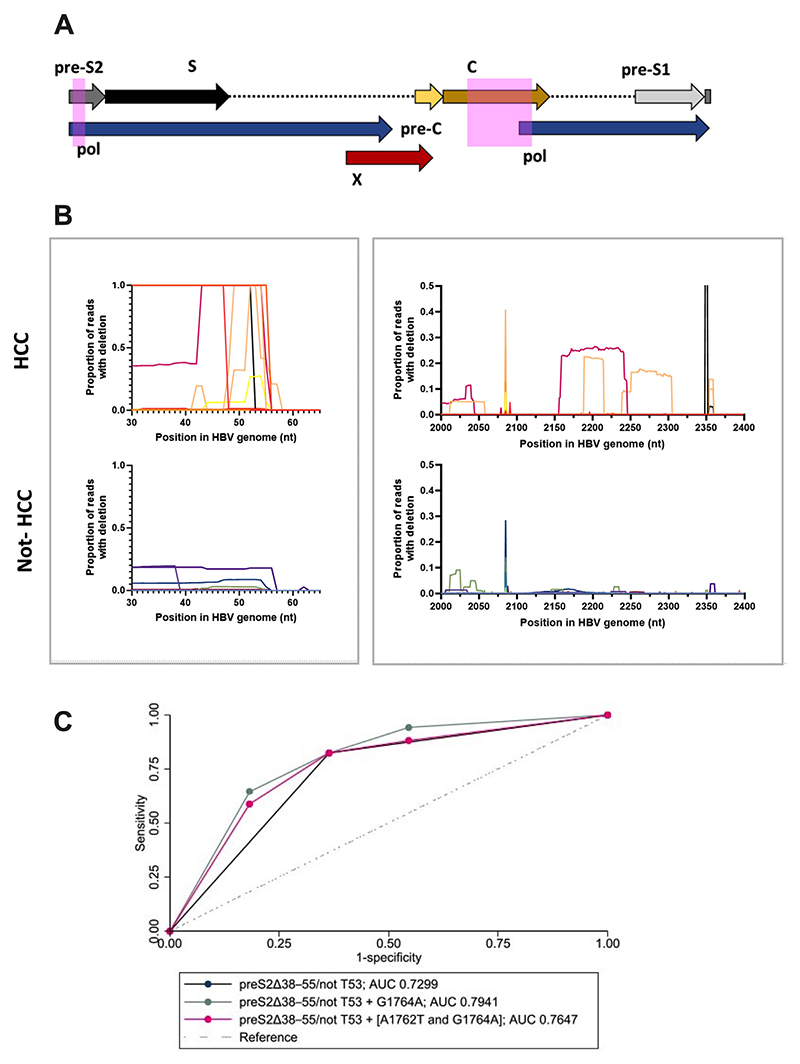
HBV Sequence motifs associated with HCC in South African adults. A: Schematic of HBV genome. Region of PreS2 and BCP polymorphisms are shown in pink highlights. B: Regions of the HBV genome showing the proportion of reads with deletions at each site. Data are presented for individuals with HCC (top) and without HCC (bottom). Termination of deletions in 7 HBV sequences from the HCC group was at nt 52 (*n* = 1), nt 53 (*n* = 2), nt 54 (*n* = 2), and nt 55 (*n* = 2). Additional discussion of the minority variant deletions is provided in Suppl results 2. The bioinformatic pipeline did not provide deletion frequencies for reads when a deletion was the consensus at that site (the site was absent), so these sites have been assigned a frequency of 100 % to generate these plots. C: Receiver operating characteristic (ROC) curves for pre-S2 deletions and T53 polymorphisms combined with BCP polymorphisms as a predictor of HCC status in HBV sequences. Curves are shown for combinations of PreS2 ‘non T53’ alone, or combined with the BCP polymorphisms G1764A and the double mutation A1764T /G1764A. Two sequences, HCC-34 and nHCC-12 were excluded from the analysis as there was no sequence for the region under consideration. AUC, area under the curve.

We examined HBV sequences for the presence of polymorphisms and deletions previously associated with HCC (Table 1). There was sequence coverage in the PreS2 region in 17 of 19 HCC cases, among which 7 of 17 had PreS2 Δ38−55 deletions, compared to none in the non-HCC group (*p* = 0.02) (Fig. 1A, B). In the consensus sequences, deletions were observed ranging from 3 bp to 42 bp in length, with a mean of 21 bp. Start locations of the sequences varied, but all deletions terminated by nucleotide (nt) 55. We also evaluated the T53C substitution that has been reported in association with HCC [30]. In the HCC group, wild-type T53 was uncommon, due to a combination of deletions (*n* = 6), and substitutions (*n* = 9; T53C substitution in 8 and T53 G in one). Thus, overall, the ‘non-T53’ motif occurred in 15/19 samples in the HCC group compared to 4/11 in the non-HCC group (*p* = 0.027; Table 1).

### 3.3 BCP mutations

Mutations in the BCP region associated with HCC by other studies (A1762T, G1764A, and the combination of both mutations) were more common in the HCC group; however, this was not significantly different from the non-HCC group (Table 1).

#### 3.3.1 Associations of combined polymorphisms with HCC

Finally, we analysed ‘non-T53’ together with the most common BCP mutation (G1764A) or the BCP double mutation (A1762T + G1764A). The combination of ‘non-T53’ with the G1764A mutation was the most strongly associated with HCC (AUROC = 0.79) (Table 1, Fig. 1C).

## 4 Discussion

In this small retrospective sample set, HBV PreS2 ‘non-T53’ (either as a result of a mutation or deletion) in combination with the BCP G1764A polymorphism, is the sequence motif most strongly associated with HCC. Both of these changes have been reported independently in South African HBV sequence data [31–35]. However, to the best of our knowledge, this combined motif has not been previously studied. Further data are needed to evaluate the sensitivity and specificity of the association between this motif and HCC.

In this small cohort, we could not determine the impact of potential confounders such as age, sex, HBV VL, HIV status, and antiviral treatment, Furthermore, there may be an impact of additional contributors which were not measured, such as host genetic/epigenetic factors, family history, coinfection with other blood-borne viruses, metabolic liver disease, obesity, diet, alcohol, and external environmental factors such as toxin exposure [36–39].

PreS2 deletions were observed in HBV sequences from people presenting with HCC in our cohort, and have also been reported in association with HCC in West African sequence data, potentially associated with aflatoxin exposure and/or conferring a viral fitness advantage [40]. Mechanistically, ‘non-T53’ may have an influence on PreS2 function or regulation and/or impact the spacer domain of the polymerase protein. Proposed oncogenic mechanisms associated with these motifs include the accumulation of defective proteins in the endoplasmic reticulum (ER), stress responses resulting in DNA damage, centrosome over-duplication, and genomic instability [28,41–43]. PreS2 deletions start at a wide range of sites (sometimes prior to nt 38) but consistently terminate immediately prior to nt 55, where there is a highly conserved region of approximately 20 bp, and the first of several cysteine residues within a putative zinc finger domain, considered to be essential for reverse transcriptase activity [44]. Therefore, deletions downstream of nt 55 are likely to be detrimental to viral replication, potentially explaining why they all terminate within the same region.

A longer duration of infection may be needed for cumulative BCP mutations to develop, and an increased frequency of these polymorphisms has been reported as HBV infection progresses [45,46]. The increase in A-T-rich regions in the BCP region (nt 1762−1770) seen in HCC-associated sequences in this study may be associated with upregulating viral transcription [47], and these mutations may influence malignant transformation mediated through the overlapping *X* gene [19].

In future, larger studies are needed, supported by an unbiased, sequence-agnostic approach to WGS changes (instead of focusing only on polymorphisms that have been previously reported). There is a need to interrogate the impact of HIV coinfection, as this may affect oncogenic risk through its effect on inflammation and immune responses (although, conversely, people living with HIV may derive protection from earlier and more consistent treatment [48]). Longitudinal follow-up in a multi-centre study would be needed to determine whether relevant HBV mutations pre-date HCC, or evolve as cancer develops.

To date, HBV sequencing protocols have been limited by high VL thresholds and/or cost. As sequencing only generates data for VL samples above a certain threshold, there may be bias in our dataset, e.g. with artificial enrichment for A1762T and G1764A mutations (lower VL samples are not represented). Retrieval of whole or partial viral sequences is also influenced by variables including quality and quantity of stored material, extent of host DNA contamination, and potentially viral genotype. As the sensitivity and efficiency of pan-genotypic sequencing methods improves, it may be possible to sequence HBV genomes at lower VL from smaller sample volumes, and to generate long-read sequences to support WGS haplotype analysis [23].

## 5 Conclusions

In conclusion, we propose a novel HBV sequence motif that may be associated with HCC, and highlight the pressing need for the field to be expanded by the open-source sharing of HBV sequences together with clinical metadata. Further work is required to determine the cause, effect and chronology of these sequence changes relative to clinical and demographic characteristics, and the evolution of HCC.

## Supplementary Material

Supplementary material associated with this article can be found in the online version at doi:10.1016/j.aohep.2024.101763.

Supplementary file

## Figures and Tables

**Table 1:** Frequency of HBV polymorphisms previously associated with hepatocellular carcinoma (HCC) identified in consensus sequences derived by Illumina among adults with and without a diagnosis of HCC. Frequencies are based on analysis of consensus sequences. Partial genomes were derived in some cases; hence, the denominator at certain sites is lower than the total number of samples sequenced.

HBV genomic region and amino acid site	Frequency of HBV polymorphism in HCC (*n* = 19)	Frequency of HBV polymorphism in non-HCC (*n* = 12)	p-value
HBsAg polymorphisms and deletions [30,40,49,50]			
W172*	0/19	0/12	n/a
W182*	0/19	0/12	n/a
L216*	1/19	0/11	0.63
Pre-S2T53C (F22L)	8/18	3/11	0.30
PreS2Δ38–55^a^	7/17	0/11	0.02
‘Non-T53’ (Pre-S2 T53C/A/G OR Δ38–55^b^)	15/19	4/11	0.03
Other pre-S1 deletion	0/17	0/11	n/a
Other S deletion	0/19	0/11	n/a
Pre-core region
Q2*	1/19	0/12	0.61
S13T	12/19	8/12	0.58
W28* (also reported as G1896A)	5/19	2/12	0.44
G29D *(also reported as G1899A)*	3/19	1/12	0.49
BCP region[14], overlapping with X protein changes
T1753C *(I127T in X protein)*	4/19	1/11	0.38
A1762T *(K130Min X protein)*	12/18	4/11	0.11
G1764A *(V131I in X protein)*	14/18	5/11	0.09
C1766T *(F132Y/I/R*^c^ *in X protein*)	6/18	1/11	0.15
T1768A	4/19	1/11	0.38
Double mutation A1762T/G1764A	11/18	4/11	0.18
Triple mutation T1753C/A1762T/G1764A	3/18	0/11	0.22
Deletion spanning1762–1764	1/19	0/11	0.63

* Stop mutation indicated by.

a The presence of any deletion spanning the region preS2Δ38−55 was recorded [40].

b The preS2 Δ38−55 deletion and the T53C mutation spanned the same site so were analysed together and considered as ‘non T53’.

c F132Y was the only variant observed at site 132 in our sequences.

## Data Availability

Illumina reads and *de novo* assemblies are available from the European Nucleotide Archive (GenBank project PRJEB71107). Consensus sequences are also available on line at https://www.doi.org/10.6084/m9.figshare.24874557.
